# Human Folate Bioavailability 

**DOI:** 10.3390/nu3040475

**Published:** 2011-04-18

**Authors:** Veronica E. Ohrvik, Cornelia M. Witthoft

**Affiliations:** 1 Science Department, National Food Administration, P.O. Box 622, SE-75126 Uppsala, Sweden; Email: Veronica.Ohrvik@slv.se; 2 Department of Food Science, Swedish University of Agricultural Sciences, Uppsala BioCenter, P.O. Box 7051, SE-75007 Uppsala, Sweden

**Keywords:** folate, folic acid, human bioavailability, intervention trials, post-dose plasma kinetics

## Abstract

The vitamin folate is recognized as beneficial health-wise in the prevention of neural tube defects, anemia, cardiovascular diseases, poor cognitive performance, and some forms of cancer. However, suboptimal dietary folate intake has been reported in a number of countries. Several national health authorities have therefore introduced mandatory food fortification with synthetic folic acid, which is considered a convenient fortificant, being cost-efficient in production, more stable than natural food folate, and superior in terms of bioavailability and bioefficacy. Other countries have decided against fortification due to the ambiguous role of synthetic folic acid regarding promotion of subclinical cancers and other adverse health effects. This paper reviews recent studies on folate bioavailability after intervention with folate from food. Our conclusions were that limited folate bioavailability data are available for vegetables, fruits, cereal products, and fortified foods, and that it is difficult to evaluate the bioavailability of food folate or whether intervention with food folate improves folate status. We recommend revising the classical approach of using folic acid as a reference dose for estimating the plasma kinetics and relative bioavailability of food folate.

## 1. Introduction

The preventive effect of high folic acid intake against neural tube defects (NTD) is considered one of the most important nutritional discoveries in the period 1976-2006 [1]. Folate requirements are increased in life stages with amplified cell division, e.g., during pregnancy, as folate is essential during cellular replication. It is assumed that on a population level, nutritional requirements for folate, as with those for vitamin D, cannot be completely covered by a “varied diet”, as recommended by national health authorities [2]. Dietary intake is below recommendations [3] in various Western societies owing to low consumption of folate-rich foods, e.g., pulses, citrus fruits, and leafy vegetables. It is estimated that an additional intake of 50-180 µg folate would allow most to reach the recommendations [4]. Populations of low socio-economic status have limited access to folate-rich foods, and furthermore natural food folates are rather unstable molecules, so that average losses in vitamin activity of around 30% can be expected during food processing [5]. However, the only risk factor for NTD which can be directly affected is inadequate dietary folate intake [6].

The chemically most stable folate form is synthetic folic acid [7], which is cheap to produce and therefore used for dietary supplements and food fortification. The introduction of folic acid-fortified staple foods has effectively decreased the prevalence of NTD, e.g., in the USA and Canada [8]. Significantly improved stroke mortality has also been reported [9]. In most countries, the Recommended Dietary Allowance (RDA) for folate is 300 µg/day for adults and 400 µg/day for women of childbearing age. With respect to vascular function, a folate intake according to the above recommendations has been shown to be beneficial, whereas higher doses do not provide any further health benefits [10]. The recommendations are more easily met by consumption of folic acid-fortified foods, owing to the higher stability and bioavailability of synthetic folic acid compared with native food folates [11]. The bioavailability of food folate is commonly estimated at 50% of folic acid bioavailability when establishing food recommendations [2,12], but this should be considered a rough estimate, as data on the bioavailability of food folate vary between 30% [13] and 98% [14]. In the US, dietary recommendations are expressed in terms of Dietary Folate Equivalents (DFE) to account for differences in the bioavailability of native food folate and folic acid fortificant [12]. The discrepancies in folate bioavailability estimates are caused by the nature of the test food or meal (as thoroughly reviewed [15,16]), entrapping the vitamin and affecting stability and absorption, inter-subject variation, different study designs and, as recently suggested, metabolic inequities between different folate forms with respect to post-absorption plasma kinetics in the human metabolism [17,18,19]. Another important aspect is that the interpretation of bioavailability data is hampered by large methodological differences during folate quantification in clinical samples and test foods, which is complicated by lack of standardized reference methods for sample preparation and folate quantification, internal standards, and use of certified reference material [20,21,22]. Recent publications question the validity of the conventional approach used in bioavailability studies comparing folic acid with natural food folate [19], and demand approaches using whole diets rather than single foods [11] or data from meta-analyses of controlled feeding trials [23] in order to closer elucidate post-absorptive metabolism of the different folate forms. This is considered necessary for evaluating the adequacy of US RDA for folate [23], and also for estimating potential risks from mandatory folic acid fortification, as a high intake of synthetic folic acid is suggested to accelerate the development of, e.g., colorectal cancer [24,25,26]. 

## 2. Synopsis of Post-Absorptive Folate Metabolism

It is commonly assumed that food folates, which mainly exist in their polyglutamyl form, are absorbed in the jejunum as monoglutamyl folates after removal of the polyglutamyl chain by intestinal γ-glutamyl hydrolase [27], and thereafter reduced and methylated in the enterocyte. Passive diffusion across the cell membrane is limited [28], and occurs only at high doses. To a minor extent folate is also absorbed in the colon; and it is suggested that colonic absorption may contribute significantly to total folate absorption [29], but it is unknown how relevant this is for maintaining folate status. However, it has been shown for humans [29,30] and pigs [31] that folates synthesized by colon bacteria are bioavailable. Absorbed folate is transported to the liver, which contains about half the body pool of folate [32,33] and retains 10-20% of absorbed folate due to the first-pass effect [15], while the rest is transported via the systemic circulation to body tissues. Some liver folate participates in the enterohepatic circulation and is secreted into bile [33,34]. However, most biliary folate is reabsorbed, supposedly to moderate between-meal fluctuations in folate supply to cells [34]. 

**Figure 1:**
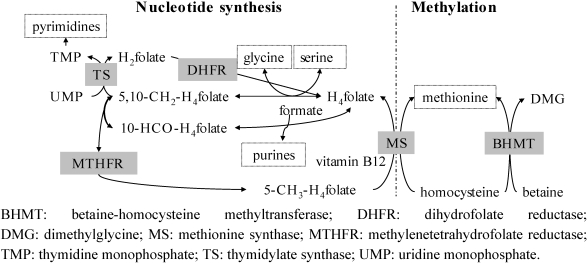
Role of folate in nucleotide synthesis and the methylation cycle (adapted from [37]). Enzymes are shaded and their end-products marked with boxes. Adapted from [38], p. 19.

In the cell nucleus, tetrahydrofolate-polyglutamate (H_4_-folate) is the substrate for both nucleotide synthesis and the methylation cycle (Figure 1), by providing or accepting one-carbon atoms [35]. 5-methyltetrahydrofolate (5-CH_3_-H_4_folate) donates a methyl group to vitamin B12, which transfers it to homocysteine, avoiding accumulation of homocysteine in the cell and maintaining synthesis of amino acids. The first signs of deficiency appear in tissues with rapid cell turnover and during life stages with increased cell division, such as pregnancy or growth [36], and can result in megaloblastic anemia by impaired synthesis of red blood cells [33]. 

It is estimated that about 0.3-0.8% of the folate pool is excreted daily, mainly in the form of catabolites not affected by intake [32,39]. Renal excretion of intact folate is as low as ~5% after physiological doses [34,40,41,42], but it increases at higher folate intake [37]. In human feces, about 400 nmol/day folate can be found, originating mainly from bacterial production, lysed enterocytes, and gastrointestinal secretions (e.g., bile) [34,43]. Little to at most 20% of fecal folate is estimated to derive from non-absorbed food folate [34,44,45,46,47]. 

Genetic polymorphisms of key enzymes and carriers can affect cellular folate metabolism and result in decreased enzyme activity, increased folate requirements [48,49,50], and an increased risk of NTD [51]. Furthermore, vitamin B12 deficiency reduces the activity of methionine synthase (MS; Figure 1), resulting in cellular accumulation of homocysteine and 5-methyltetrahydrofolate according to the methyl trap theory [37]. 

## 3. Estimation of Folate Bioavailability-Still a Challenge

Accurate estimation of dietary folate bioavailability is a prerequisite for the formulation of nutrition recommendations, but is still a challenge. Folate absorption is defined as the *in vivo* process by which folate proceeds from the site of administration to the site of measurement (usually plasma) [52]. Folate bioavailability is defined as the fraction of ingested folate that is absorbed and can be used for metabolic processes [16]. Findings from early bioavailability studies until the mid-1990s discussed by others indicate the need for further investigations on folate bioavailability and influencing factors [53].

Long-term (Table 1 and Table 2) and short-term (Table 3) human trials, conducted during the past 15 years, have produced inconclusive findings regarding dietary folate bioavailability, complicating the interpretation of data by different definitions for the term bioavailability and differing study designs. Furthermore, difficulties with respect to the exact quantification of ingested folate dose from food samples and in clinical samples limit the validity of many studies [19,54]. Although there are recommendations for the extraction of food samples using trienzyme treatment [55,56,57], this approach is used in only some studies [13,14,45,58,59,60,61,62,63,64]. Others apply dienzyme [46] or monoenzyme [18,40,42,65] extraction prior to folate quantification by microbiological assay or HPLC (LC-MS or LC-FLD). Information is sometimes missing [17,66,67], or dietary folate doses are determined by calculation [19,68,69,70] (Table 1 ,and ).

**Table 1:** Dietary folate intervention trials resulting in no significant effects (presented as change (%) compared with baseline) on folate status.

Intervention diet	Folate intake		W	*N*	Age	Erythrocyte folate		p/s-folate		tHcy (µmol/L)		Ref.
	Dose (µg/day) (Q) ^1^	Total ^2^(µg/day)				Baseline(nmol/L)	End(%)	Baseline(nmol/L)	End(%)	Baseline(nmol/L)	End(%)	
Control	2 (C) ^1^		4	35, F	41 ± 3 ^3^			15 ± 9 ^3^	−15	11 ± 4 ^3^	−10	[68]
5-a-day fruit/veg	43 (C) ^1^		4	36, F	42 ± 4 ^3^			17 ± 9 ^3^	−6	10 ± 3 ^3^	0	
Control	0 (T) ^1^	186	4	18, M	23-39 ^4^			16 ± 9 ^3^	−7	12 ± 4 ^3^	0	[13]
Spinach	200 (T) ^1^	384	4	18, M	23-39 ^4^			14 ± 5 ^3^	−7	12 ± 3 ^3^	0	
Yeast	200 (T) ^1^	411	4	19, M	23-39 ^4^			15 ± 7 ^3^	−17	12 ± 3 ^3^	−9	
Control	23 (L) ^1^	210	12	9, F	17-40 ^4^	709 ± 157 ^3,5^	+3 ^5^					[66]
High folate diet	201 (L) ^1^	410	12	10, F	17-40 ^4^	797 ± 200 ^3,5^	+7 ^5^					

F: females; M: males; W: weeks; *N*: number of subjects; Veg: vegetables; p/s-folate: plasma/serum folate concentration; tHcy: total homocysteine concentration in plasma/serum; End (%): relative increase (+) or decrease (−) in status parameter at end of intervention compared with baseline. ^1^ Q: Quantification of folate content in intervention doses by (C: calculation, T: using trienzyme extraction, L: lacking information); ^2^ Estimated by dietary recall (either reported as total folate intake or estimated as dose + folate intake at screening); ^3^ Mean ± SD; ^4^ Age reported as inclusion criteria; ^5^ Erythrocyte folate concentrations given in µg/L, calculated to nmol/L (0.460^−1^ nmol/µg).

**Table 2:** Dietary folate intervention trials resulting in significant effects (presented as change (%) compared with baseline) on folate status.

Intervention diet	Folate intake (µg/day)		W	*N*^2^	Age	Erythrocyte folate		p/s-folate		tHcy		Ref.
	Dose (Q) ^1^	Total				Baseline (nmol/L)	End (%)	Baseline (nmol/L)	End (%)	Baseline (µmol/L)	End (%)	
Control	0	236	16	43	-			26 ± 8 ^3^	−4	9 ± 3 ^3^	0	[69]
5-a-day fruit/vegetables	63 (C)	306	16	41	-			26 ± 7 ^3^	+7 ^♦^	10 ± 4 ^3^	−11	
Low folate diet	0 (L)	131	4	23	50 ± 4 ^3^			13 ± 6 ^3^	−8	14 ± 6 ^3^	−8	[67]
500 g fruit and vegetables	97 (L)	228	4	24	49 ± 6 ^3^			16 ± 10 ^3^	0 ^♦^	12 ± 4 ^3^	−9 ^♦^	
Control	0	239	12	17, F	48 (38, 57) ^7^	900 (647, 1079) ^7^	−22 ^7^	12 (11, 15)	0 ^7^	9 (7, 11)	+10 ^7^	[59]
5 slices bread	70 (T)	379	12	17, F	47 (40, 55) ^7^	855 (635, 992) ^7^	−18	12 (9-16)	0	9 (8, 11)	−21 *	
Breakfast (SNO [71])	125 (T)	437	12	17, F	49 (35, 53) ^7^	805 (742, 909) ^7^	+12 ^♦^	12 (10, 16)	+8	7 (7, 9)	−23 *^,♦^	
Folic acid-fortified bread	188 (T)	461	4	31	36 ± 13 ^3^	606 ± 224 ^3^	+13 *	12 ± 5 ^3^	+25 *			[60]
Rye and orange juice	184 (T)	461	4	33	36 ± 13 ^3^	561 ± 190 ^3^	+15 *	10 ± 3 ^3^	+23 *			
Folic acid	150 (T)	221 ^4^	4	15	19-48 ^5^			12 ± 4 ^3^	+20	10 ± 2 ^3^	−11	[61]
Vegetables, fruits, liver pate	331 (T)	427 ^4^	4	29	19-41 ^5^			12 ± 4 ^3^	+33 ^♦^	10 ± 3 ^3^	−11	
Placebo	0	~360 ^9^	16	43	46 ± 2	895 ±6 0	+3	26 ±2	−1	9 ± 0.4	0	[19]
Food group	200 (C)	~305 ^9^	16	38	45 ± 2	872 ± 61	+14 ^♦^	25 ± 2	+19 ^♦^	10 ± 1	−6 ^♦^	
5-methyltetrahydrofolate caps	200 (P)	~295 ^9^	16	42	45 ± 2	793 ± 42	+30 ^♦^	22 ± 2	+41 ^♦^	10 ± 1	−14 ^♦^	
Folic acid caps	200 (P)	~325 ^9^	16	40	48 ± 2	833 ± 56	+28 ^♦^	24 ± 2	+44 ^♦^	9 ± 0.4	−15 ^♦^	
Control	0	210 ^4^	4	22	23 ± 8 ^3^	347 ± 79^3^	−1	13 ± 3 ^3^	0	10 ± 3^3^	+9	[14]
Fruit and vegetables	350 (T)	560 ^4^	4	23	23 ± 8 ^3^	338 ± 81^3^	+16 ^♦^	14 ± 3 ^3^	+30 ^♦^	11 ± 5^3^	−10 ^♦^	
Control	0 (T)	242	4	14	60 ± 15 ^3^			18 (15-22) ^6^	0 ^6^	14 (12-16) ^6^	0 ^6^	[62]
High folate diet	355 (T)	618	4	20	58 ± 18 ^3^			18 (16-19) ^6^	28 ^6,^^♦^	12 (11-13) ^6^	−9 ^6,♦^	
Control	0 (T)	227	12	15	36-71 ^8^	539 ± 166 ^3^	0	15 (12-18) ^6^	−7 ^6^	12 (11-14) ^6^	0 ^6^	[58]
High folate diet	~350 (T)	707	12	15	36-71 ^8^	571 ± 162 ^3^	+11	15 (13-17) ^6^	+32 ^6,♦^	11 (9-12) ^6^	−10 ^6^	
Pericarp flour bread	223 (T)	436	16	25	48-56 ^5^	497 (414-581) ^6^	+14 ^6^	13 (11-15) ^6^	+7 ^6^	10 (8-11) ^6^	−25 ^6^	[63]
Aleurone flour bread	615 (T)	836	16	25	46-54 ^5^	509 (434-584) ^6^	+34 ^6,♦^	13 (10-16) ^6^	+52 ^6,♦^	9 (8-10) ^6^	−29 ^6,♦^	

W: week; caps: capsules; *N*: number of subjects; F: females; p/s-folate: plasma/serum folate concentration; tHcy: total homocysteine concentration in plasma/serum. End (%): relative increase (+) or decrease (−) in status parameter at end of intervention compared with baseline. ^1^ Q: Quantification of folate content in intervention doses by (C: calculation, T: using trienzyme extraction, L: lacking information); P: as provided by manufacturer; ^2^ Females and males; ^3^ Mean ± SD; ^4^ Folate dose analyzed and not estimated by dietary recall; ^5^ Range; ^6^ Geometric means (95% CI); ^7^ Median (quartile 1, quartile 3); ^8^ Age reported as inclusion criteria; ^9^ Folate intake given in nmol, calculated to µg (0.460^−1^ nmol/µg). * Significant effect within the group compared with baseline (*P* < 0.05); ^♦^ Significant effect compared with control (*P* < 0.05).

**Table 3:** Trials using plasma concentration to assess the bioavailability of food folates or fortificants.

	Dose nmol ^1^	AUC or App Abs ^2^	Sampling duration (nr/4 h) ^3^	Females + males	Age ^4^	Ref.
Size of AUC (h∙nmol/L) from supplements and fortified foods						
[13C6]-folic acid	634	19	8 h (8)	10 ^10^	31 ± 1	[17]
Folic acid 5	1134	146	10 h (6)	0 + 13	26 ± 6	[64]
Folic acid	1134	40	10 h (6)	6 + 6	36-69	[45]
Folic acid	1193	37, 49 ^7^	7 h (3)	8 + 8	20-50	[65]
Folic acid	907	62	10 h (4)	10 + 10	27 ± 3	[70]
[13C5]-folic acid in bread	450 (D)	28	12 h (7)	5 + 3	39-66	[46]
[13C5]-folic acid in breakfast meal	450 (D)	26	12 h (7)	5 + 3	39-66	[46]
(6S)-[13C6]-5-HCO-H4folate	500	42	8 h (8)	10 ^10^	31 ± 1	[17]
(6*S*)-5-CH_3_-H_4_folate ^5^	1088	142	10 h (6)	0 + 13	26 ± 6	[64]
(6*S*)-5-CH_3_-H_4_folate ^5^	830 ^6^	44, 88 ^6^	10 h (7)	0 + 2	55, 77	[40]
(6*S*)-5-CH_3_-H_4_folate i.m. ^5^	830 ^6^	111, 144 ^6^	10 h (7)	0 + 2	55, 77	[40]
(6*S*)-[13C5]-5-CH_3_-H_4_folate in bread	450 (D)	66	12 h (7)	5 + 3	39-66	[46]
Size of AUC (h∙nmol/L) from native food folates						
Broccoli ^5^	440 (M)	27, 41 ^6^	10 h (7)	0 + 2	55, 77	[40]
Strawberries ^5^	450 (M)	32, 41 ^6^	10 h (7)	0 + 2	55, 77	[40]
Spinach, monoglutamate ^8^	820 (T)	31	10 h (6)	6 + 6	36-69	[45]
Spinach, polyglutamate ^9^	990 (T)	27	10 h (6)	6 + 6	36-69	[45]
Spinach	544 (C)	41	10 h (4)	10 + 10	27 ± 3	[70]
	1088 (C)	71				
Aleurone flour	1167 (M)	46, 38 ^7^	7 h (3)	8 + 8	20-50	[65]
Wheat bran	213 (M)	8, 6 ^7^	7 h (3)	8 + 8	20-50	[65]
Apparent absorption (%) from supplements, fortified foods and food folate						
[13C6]-folic acid	634	24%	8 h (8)	14 ^10^	33 ± 2	[18]
(6S)-[13C6]-5-HCO-H4folate	500	38%	8 h (8)	14 ^10^	33 ± 2	[18]
Spinach [15N1-7]folate	588 (M)	44%	8 h (8)	14 ^10^	33 ± 2	[18]
Folic acid in bread ^5^	491 ^6^ (M)	74%	10 h (7)	1 + 8	51-79	[44]
(6*S*)-5-CH_3_-H_4_folate in fermented milk ^5^	450 ^6^ (M)	86%	10 h (7)	1 + 8	51-79	[44]
Yeast folate ^5^	155 ^6^ (M)	80%	10 h (7)	1 + 8	51-79	[44]

5-HCO-H_4_folate: 5-formyltetrahydrofolate; 5-CH_3_-H_4_folate: 5-methyltetrahydrofolate; [13C5]-, [13C6]-: stable isotope-labeled (5 or 6 ^13^C); (6*S*)-: bioactive folate diastereoisomer; ^1^ Folate content in food intervention doses quantified by (T: trienzyme, D: dienzyme or M: Monoenzyme extraction, or by C: calculation); ^2^ App abs: apparent absorption, estimated by (kinetic modeling [18,42] or AUC: area under the curve (Mean or Median)); ^3^ Number of sampling occasions during 0-4 h post-dose in brackets; ^4^ Mean ± SD or range; ^5^ Presaturation with ~1 mg [38,42] or 5 mg [72] folic acid/day from day −9 to day −2 prior to each test day; ^6^ Conversion factors from µg to nmol: 0.441^−1^ nmol/µg (folic acid) and 0.460^−1^ nmol/µg (plasma folate and 5-methyltetrahydrofolate);^7^ Females and males reported separately; ^8^ Folate monoglutamates from enzymatic cleavage of endogenous spinach folates after cell disruption; ^9^ Native spinach folate polyglutamates; ^10^ No data on gender.

### 3.1. Intervention Trials

Physiological effects of dietary folate interventions are commonly studied in 4-6 week trials assessing changes in one or several folate status parameters, e.g., fasting serum folate, erythrocyte folate, and plasma total homocysteine (Table 1 and Table 2). 

It seems appropriate to assess more than one status parameter, since the sensitivity of parameters and the rapidity of response varies. For example, a 4-week intervention study found the estimated bioavailability of food folate compared with folic acid to be 78% based on serum folate concentrations, 98% based on erythrocyte folate concentrations, and only 60% based on homocysteine concentrations [14]. It is recommended that the appropriateness of the chosen status parameters in intervention trials be evaluated based on reliability coefficients [73,74,75]. Reliability coefficients show the ratio of between-subject variability to total variability, including the variability of the analytical method. Reliability coefficients for the common response parameters in folate intervention studies are within the range 0.65-0.97 [38,73,74,75]. 

Interpretation of the effects of intervention on folate status and bioavailability is further complicated due to the fact that several genetic polymorphisms of folate/homocysteine metabolic enzymes may affect the individual parameters differently [72,76]. 

Several intervention trials report a low relative bioavailability of food folates compared with folic acid (Table 1 and Table 2) [13,58,66]. On the other hand, improvement of folate status parameters from food folates has been observed compared with a control group receiving no additional folate (Table 2) [59,67]. 

Data from a very recent study [19] show that the relative response of folate status parameters after food intervention differs compared with a reference group receiving supplemental folic acid or the bioactive diastereoisomer (6*S*)-5-methyltetrahydrofolate (Metafolin^®^). As the intervention with folic acid results in a greater increase in fasting plasma folate concentrations than bioactive folate diastereoisomer, the estimated relative bioavailability of food folate is lower when compared with the supplemental folic acid receiving control group. Those authors therefore recommend avoiding using folic acid as the reference dose in long-term studies. However, this hypothesis remains to be confirmed, as some intervention trials with foods have resulted in similar improvements of folate status compared with equimolar or smaller doses of folic acid from fortified foods or supplements [60,61,63].

### 3.2. Short-Term Trials

Short-term absorption or bioavailability of food folate has been found to be incomplete compared with that of folic acid [77], and as a result a number of approaches for assessment are now used. 

The plasma area under the curve (AUC) approach is based on the assumption that with standardized study design, the post-dose plasma folate concentrations over time correspond to the folate fraction absorbed from a single dose. Relative folate absorption is commonly assessed, comparing the test dose with an oral reference dose of supplemental folate, often folic acid (Table 3). Comparisons of the relative AUC after a single dose of supplemental folic acid and reduced folates show that the trials using presaturation of volunteer’s body stores [40,44,64] result in greater AUCs than others. This suggests that after administration of an oral dose, the amount of folate in peripheral plasma is affected by the hepatic first-pass effect and volunteer’s body stores, which could explain the variable results in published bioavailability data. The data are too few to systematically evaluate whether equimolar doses of folic acid and reduced folate result in differently sized plasma AUCs, as administered doses, sampling intervals, number of sampling occasions, and presaturation of volunteers vary between trials (Table 3). 

In three previous trials [18,45,70], the AUC or apparent absorption of spinach folate was estimated to be similar or greater than almost equimolar doses of synthetic folic acid, demonstrating in theory similar bioavailability. However, it was hypothesized that reduced folates and folic acid show differing plasma and distribution kinetics [17,18]. It was suggested that the initial site of folic acid metabolism is not the enterocyte, as accepted in the past, but the liver cell, and liver enzymes are postulated to have a greater affinity for oxidized folic acid than reduced folates. Therefore it is not considered appropriate to compare natural folate test doses (e.g., 5-methyltetrahydrofolate, 5-formyltetrahydrofolate) with a folic acid reference dose [18]. 

With the aim of estimating absolute folate absorption and avoiding complications from differing plasma kinetics from test and reference compounds, a reference dose of the bioactive diastereoisomer (6*S*)-5-methyltetrahydrofolate, administered by intramuscular injection to bypass the liver, was used to estimate the bioavailability of native folate from foods [40]. In this pilot trial with only two volunteers, the AUCs from intramuscularly administered (6*S*)-5-methyltetrahydrofolate were greater than those from an oral dose of the same compound (Table 3), indicating partial hepatic retention of the orally administered folate dose even when volunteers were presaturated. 

In some trials the apparent absorption accounting for the folate distribution volume in the body has been determined using biokinetic modeling, and these estimated that 24-86% of supplemental or native food folate is absorbed (Table 3) [18,44]. Some studies [78] are limited by too short post-dose plasma sampling, so that the AUC cannot be estimated and extrapolated for exact assessment of absorbed folate, or no plasma AUC data are provided [42,79]. 

The determination of folate bioavailability by urinary folate excretion ratio is based on the hypothesis that urinary excretion of [13C]-labeled folate from an oral dose corresponds to that of a differently labeled reference dose of [2H]-folic acid from analogous intravenous administration. This dual-label stable isotope protocol, when used to determine relative folate bioavailability of food folates and added fortificants, produces values of 37-153% [41,42,80,81,82]. However, these studies also generated the information that reduced folates and folic acid are handled differently by the body; and (6*S*)-5-methyltetrahydrofolate was recommended as the reference dose [80]. 

Studies using the oral-fecal balance technique for folate are commonly carried out using ileostomy subjects, who do not possess a colon microflora affecting fecal folate content, in combination with the AUC technique. Absorbed folate is inversely correlated to folate content in stomal effluent. However, not all folate, found in ileostomy effluent or feces, originates from non-absorbed orally ingested folate [43], and therefore baseline folate excretion has to be assessed. Baseline folate excretion is determined either on a folate-free study day using a standardized protocol [40,44], or after a known reference dose of synthetic folic acid [45]. The use of stable isotope labeling allows folate from the dose to be distinguished from endogenous folate [34,83]. Folate bioavailability from foods and a meal has been estimated to be 50-90% using the balance technique [30,44,45,46,84]. 

A common criticism of short-term protocols is the use of non-physiological doses, or large portions of test foods that are not likely to be consumed in a meal by the general population, e.g., 30 g of yeast [77] or 500 g of spinach [70], to overcome insufficient sensitivity of the model. To improve sensitivity and minimize or standardize hepatic retention of absorbed folate at first passage, folate body stores can be presaturated with supplemental folic acid [77] (Table 3). However, this approach can also be questioned, as it has been shown *in vitro* that high exposure to folic acid reduces cellular uptake into Caco-2 and kidney cells by down-regulation of genes coding for folate absorption and transport [85]. 

**Figure 2:**
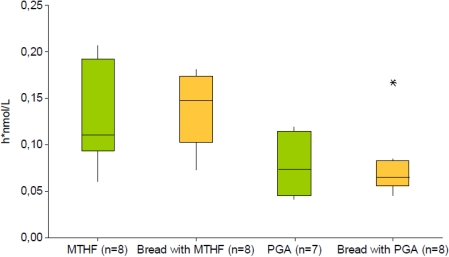
Dose-normalized AUC of plasma [13C5]5-methyltetrahydrofolate (h* nmol/L) after single oral equimolar folate doses (≈450 nmol = 200 µg) in the form of pharmaceutical preparation with (6*S*)-[13C5]5-methyltetrahydrofolate (MTHF, green) or [13C5]folic acid (PGA, green) or as bread fortified with (6*S*)-[13C5]5-methyltetrahydrofolate (bread with MTHF, orange) or [13C5]folic (bread with PGA, orange). * indicates an outlier. Data from [46,86].

A recent study uses a combined approach of various short-term techniques in the form of a stable isotope AUC and ileostomy model [46,47] to determine acute absorption of equimolar doses of either stable isotope-labeled (6*S*)-[13C5]5-methyltetrahydrofolate or [13C5]folic acid from bread or a breakfast meal (Table 3). The plasma AUC of labeled folate after ingestion of the bread fortified with bioactive (6*S*)-[13C5]5-methyltetrahydrofolate was twice that for food labeled with folic acid, whereas the excretion of labeled folate into ileostomy effluent did not differ significantly and was as low as 10% of the dose, indicating high bioavailability providing that no severe oxidative degradation of non-absorbed labeled folate occurred in frequently sampled ileostomy effluent. For supplemental (6*S*)-[13C5]5-methyltetrahydrofolate too, a greater median plasma AUC was observed compared with [13C5]folic acid [86] (Figure 2).

These findings confirm the hypothesis of different metabolic handling of reduced folates and folic acid in the human body. The consequence is a recommendation to revise the classical approach of using folic acid as the reference dose for estimation of the plasma kinetics and relative bioavailability of food folate. 

Even when the use of stable isotope-labeled folates (Table 3) offers the advantage of distinguishing between labeled folate from the dose and unlabeled endogenous folate [17,19,42,46], no data on absorption of native food folates are obtained unless intrinsic labeling is used [18]. 

Current short-term folate bioavailability data are therefore limited to a few vegetables, fruits, cereal products, and fortified foods (Table 3) [18,40,45,65,70,78] and have to be interpreted with caution.

## 4. Conclusions

This summary of existing information shows that only limited folate bioavailability data are available for vegetables, fruits, cereal products and fortified foods. It also shows the difficulties in assessment. No safe conclusion can be drawn as to whether intervention with food folate results in improved folate status. Additionally, it is not possible to estimate the size of the required minimum intervention dose of food folate to achieve improvement of status, as the status parameters are affected to different extents. 

However, the data highlight differences in the metabolic handling of folic acid and reduced folates in humans, as evidenced by differing acute post-absorptive plasma kinetics and different effects on fasting plasma folate concentrations after long-term intervention. It is still unclear whether these findings have implications for human health, and they need to be considered in recommendations regarding folic acid fortification of food. As it is not expected that the more expensive and less stable bioactive diastereoisomer (6*S*)-5-methyltetrahydrofolate (Metafolin^®^) will be commonly used for fortification purposes, synthetic folic acid reference doses are appropriate for evaluation of the effectiveness of folic acid-fortified foods at population level.

An immediate consequence is our recommendation to revise the classical approach of using folic acid as the reference dose in estimating the plasma kinetics and relative bioavailability of food folate. The design of future studies on relative folate bioavailability faces the challenge of accounting for the bias from metabolic handling of folate compounds, and existing findings have to be interpreted with caution. 
